# Association between diet quality, dietary patterns and cardiometabolic health in Australian adults: a cross-sectional study

**DOI:** 10.1186/s12937-018-0326-1

**Published:** 2018-02-12

**Authors:** Katherine M. Livingstone, Sarah A. McNaughton

**Affiliations:** Institute for Physical Activity and Nutrition, School of Exercise and Nutrition Sciences, Deakin University, Melbourne Burwood Campus, 221 Burwood Highway, Geelong, Victoria 3125 Australia

**Keywords:** Diet quality, Dietary patterns, Reduced rank regression, Cardiometabolic health, Adults

## Abstract

**Background:**

Diet quality indices score dietary intakes against recommendations, whereas dietary patterns consider the pattern and combination of dietary intakes. Studies evaluating both methodologies in relation to cardiometabolic health in a nationally representative sample are limited. The aim of the present study was to investigate the relationship between diet quality, dietary patterns and markers of cardiometabolic health in Australian adults.

**Methods:**

Dietary data, using two 24-h dietary recalls, were collected from adults in the cross-sectional Australian Health Survey 2011–2013 (*n* = 2121; 46.4 (SE 0.48) years). Diet quality was estimated using the Dietary Guideline Index (DGI). Dietary patterns (DPs), derived using reduced rank regression, were estimated using fiber density, SFA: PUFA and total sugars intake as intermediate markers. Multi-variable adjusted linear regression analyses were used to examine associations between diet quality and DPs and blood biomarkers, body mass index, waist circumference, diastolic and systolic blood pressure and an overall cardiometabolic risk score.

**Results:**

DGI was associated with lower glucose (coef − 0.009, SE 0.004; P-trend = 0.033), body mass index (coef − 0.017, SE 0.007; P-trend = 0.019) and waist circumference (coef − 0.014, SE 0.005; P-trend = 0.008). Two dietary patterns were derived: dietary pattern-1 was characterized by higher intakes of pome fruit and wholegrain bread, while dietary pattern-2 was characterized by higher intakes of added sugars and tropical fruit. Dietary pattern-1 was associated with lower body mass index (coef − 0.028, SE 0.007; P-trend< 0.001) and waist circumference (coef − 0.017, SE 0.005; P-trend = 0.001). There was a trend towards lower cardiometabolic risk score. Dietary pattern-2 was associated with lower HDL-cholesterol (coef − 0.026, SE 0.012; P-trend = 0.028). There was a trend towards lower diastolic blood pressure. No associations with other markers were observed.

**Conclusions:**

Better diet quality and healthier dietary patterns were primarily associated with favorable anthropometric markers of cardiometabolic health. Findings support the need for comparison of whole-diet based methodologies that take into consideration the interactions between foods and nutrients. Longitudinal studies are warranted to better understand causal relationships between diet and cardiometabolic health.

**Electronic supplementary material:**

The online version of this article (10.1186/s12937-018-0326-1) contains supplementary material, which is available to authorized users.

## Background

Non-communicable diseases, such as diabetes and cardiovascular disease, are responsible for 40 million deaths per year worldwide [1]. The development of these conditions is mediated through complex biological pathways, such as elevated blood pressure and levels of triglycerides (TAG), total and LDL cholesterol and glucose [2]. With the global health burden of poor cardiometabolic health increasing, a better understanding of major modifiable risk factors, including diet, is needed [3–5].

Research to date has focused primarily on the role of single nutrients and foods in relation to cardiometabolic health [3, 6, 7]. However, given that food and nutrient intakes are often correlated, an increasing body of research is investigating whole diet [8] and its impact on disease risk [9–11]. Whole diet analyses have traditionally been based on data-driven techniques, such as factor analysis, or diet quality indices, estimated according to adherence to dietary guidelines. By combining strengths of both methodologies, reduced rank regression (RRR) uses a priori knowledge of markers of disease risk in a posteriori dietary patterns (DP) derivation [12]. RRR is becoming increasingly used in nutritional epidemiology for deriving DPs and evaluating associations with health outcomes [13–16].

Evidence suggests that better adherence to diet quality indices, such as the Alternate Healthy Eating Index (HEI) [17, 18], the Mediterranean diet score [17] and the Dietary Guideline index (DGI) [19], is associated with more favorable levels of many cardiometabolic biomarkers. Findings from a sample of Australian adults showed that a DP, derived using factor analysis and characterized by high consumption of wholegrains and fruit, was associated with higher odds of having a metabolically healthy phenotype [20]. Similarly, in a study of US adults, a RRR-derived DP low in soft drinks and high in wholegrains was inversely associated with TAG [13]. Despite this evidence, few nationally representative studies have compared more than one whole diet measure and few have considered an overall cardiometabolic risk score, nor the moderating effect of body mass index (BMI) [21]. A comparison between diet quality and a novel DP methodologies is needed to strengthen the evidence base for future DP-based policy development [22, 23]. In addition, RRR may better predict risk of disease than purely data-driven DP methodologies [24, 25], yet there is a paucity of research that has examined RRR-derived DPs in relation to cardiometabolic health [26].

Therefore, the aim of this study was to examine the relationship between diet quality, using the DGI, and DPs, using RRR, and markers of cardiometabolic health, including BMI, waist circumference (WC), total, HDL and LDL cholesterol, glycated hemoglobin (HbA1c), glucose, TAG, apolipoprotein B, diastolic and systolic blood pressure and an overall cardiometabolic risk score, in the cross-sectional, nationally representative 2011–2013 Australian Health Survey (AHS) [27].

## Methods

### Subjects and study design

**Fig. 1:**
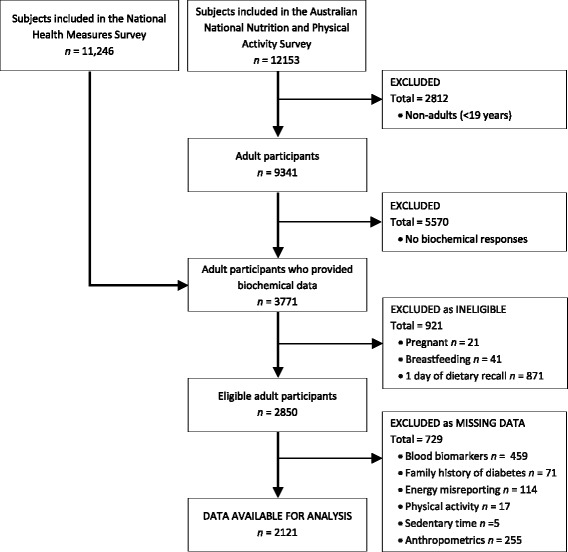
Flow diagram of subjects included in the cross-sectional analysis of the Australian Health Survey

The 2011–13 AHS is a population-based survey that sampled urban and rural households across all Australian states and territories. The AHS consists of two separate surveys (the National Health Survey [NHS] and the National Nutrition and Physical Activity Survey [NNPAS]) and the National Health Measures Survey (NHMS), a third component in which participants from both surveys were invited to participate [27]. In the NHS and NNPAS, 21,108 private dwellings (*n* = 18,355 after sample loss in the field stage) and 14,363 private dwellings (*n* = 12,366 after sample loss in the field stage) were selected, respectively. Dietary intakes were estimated in the NNPAS using two, 24-h dietary recalls. Of the 9519 dwellings in the first interview, 7735 (63.6%) completed the second dietary recall. Anthropometric and blood pressure measures were collected in the NNPAS on a voluntary basis by trained interviewers during home visits. Of the 30,329 respondents aged ≥ 5 years in the combined sample (NHS and NNPAS), 11,246 (37.1%) participated in the biomedical component (NHMS). Data relating to fasting tests relate to the fasting population only. For the present analysis individuals were excluded if they i) were pregnant and/or breastfeeding ii) only completed one day of dietary recall and iii) had missing data for outcomes and covariates (Fig. 1). A total of 2121 adults (≥ 19 y) were included in the current analysis. Ethics approval for the NHMS was granted by the Australian Government Department of Health and Ageing Departmental Ethics Committee. Further information on the design and methodologies of these surveys are presented elsewhere [27]. Reporting was conducted in accordance with the STROBE statement (Additional file 1: Table S1).

### Study measures

#### Cardiometabolic risk variables

Weight (kg), height (cm) and WC (cm) measurements were assessed on a voluntary basis by trained interviewers using digital scales, a stadiometer and a metal tape respectively. Waist measurements were taken by placing the tape measure across the top of the belly button. For validation purposes, a random 10% of respondents were selected to be measured for height and waist an additional time. If this second measurement varied by more than one cm then a third reading was taken. Weight measurements were only taken once. Subjects were encouraged to remove their shoes and any heavy clothing prior to having measurements taken, although this was not compulsory, and no correction was applied if they did not. BMI was derived using Quetelet’s metric (kg/m^2^). Standard cut offs were used to derive BMI and WC categories [28].

LDL cholesterol (mmol/L), TAG (mmol/L) and plasma glucose (mmol/L) were measured from blood samples in individuals who reporting fasting for 8 h or more prior to providing a blood sample [27]. LDL cholesterol was calculated from total cholesterol, HDL cholesterol (mmol/L) and fasting TAG levels using the Friedewald equation [29]. As recommended by the Australian Bureau of Statistics, individuals with a TAG level of ≥ 4.5 mmol/L were treated as missing data for the estimation of LDL-cholesterol [29]. Apolipoprotein B (g/L), HbA1c (mmol/mol) and total and HDL-cholesterol were measured in biological samples without the need for fasting. Abnormal levels of total, HDL- and LDL-cholesterol were defined as ≥ 5.5 mmol/L, < 1.0 mmol/L for males and < 1.3 mmol/L for females and ≥ 3.5 mmol/L, respectively. Abnormal levels of TAG and apolipoprotein B were ≥ 2.0 mmol/L and > 1.3 mmol/L for males and > 1.2 mmol/L for females, respectively. Impaired fasting plasma glucose was defined as > 6.0 mmol/L and < 7.0 mmol/L, while HbA1c levels indicative of increased risk of diabetes was defined as 42–47 mmol/mol [27]. Diabetes prevalence was estimates by self-reported diabetes diagnosed by a doctor. All analyses were conducted by Douglass Hanly Moir (Australia).

Systolic (SBP) and diastolic blood pressure (DBP) measurements were taken on the left arm. Interviewers performed two blood pressure readings using an automated blood pressure monitor. The second reading was used, unless there was a difference greater than 10 mmHg between the readings, in which case a third reading was taken and the second and third readings were averaged. Data on anti-hypertensive and lipid lowering medication were not recorded [27].

An overall cardiometabolic risk score was derived using WC, TAG, HDL-cholesterol, blood pressure (average blood pressure was used as an index for SBP and DBP), and fasting plasma glucose based on an established methodology [30]. Briefly, all variables were normalized (log 10) and standardized (i.e. z = (value − mean)/SD). For HDL-cholesterol, the z-score was multiplied by − 1. All z-scores were then summed to compile the cardiometabolic risk score with units of SD.

#### Dietary intake

A 24-h dietary recall was used to provide quantitative information on foods and beverages consumed on the day prior to interview based on the in person USDA Automated Multiple-Pass Method [31]. The interview was divided into five phases: quick list description of food and beverages consumed from midnight to midnight the previous day, prompt the respondent to remember any omitted foods, provide information on time and eating occasion, further details (including preparation method and brand names) and a final probe to recall any omitted foods of beverages [27]. A second 24-h recall, via telephone interview, was collected at least 8 days after the first interview. Only participants who completed both recalls were included in the present analysis. Nutrient intakes were derived from the 24-h recalls using the Australian Supplement and Nutrient Database 2011–13 [32]. Information on the following dietary behaviors were collected in the NNPAS survey: type of milk consumed, usual daily intake of fruit and vegetables and use of salt [27].

#### Dietary guideline index

The DGI is a food-based score designed to reflect the diet quality of individuals according to compliance with the 2013 Australian Dietary Guidelines [33]. Dietary intakes of individuals, based on an average of two 24-h recalls and brief questionnaire items, were scored according to ten recommended dietary components (food variety, fruit, vegetables, cereals [total cereals and proportion that is wholegrains], meat and alternatives [total lean meat and alternatives and proportion that is lean], dairy and alternatives and fluid intake [total beverage and proportion that is water]) and six dietary components that should be limited (discretionary foods, SFA, unsaturated fat, added salt, added sugars and alcohol). Further details are presented in Additional file 2: Table S2 and are available elsewhere [34, 35].

DGI scores ranged between 0 and 130, with a higher score indicating better diet quality. Each item was scored out of 10, with 0 indicating the guideline was not met. Cut-offs used to obtain the maximum score for each component were tailored to age- and sex- specific food-based recommendations outlined in the Australian Dietary Guidelines [36]. Proportionate scores were derived where intakes fell between the maximum and minimum scoring criteria for all items except discretionary foods, saturated and unsaturated fat, salt, sugar and alcohol, which scored either 0 or 10 [34, 37, 38].

### Dietary patterns

DPs were determined using RRR is a statistical technique designed to derive DPs that maximize the variation explained by response variables selected based on a priori hypothesis that they are related to the outcome of interest [12]. In the present analyses, fiber density, SFA: PUFA and total sugars intake were selected as response variables. These nutrients were chosen based on evidence from the WHO report on prevention of chronic disease that consumption of dietary fiber, fat and sugars are strongly associated with risk of diabetes and cardiometabolic disease [4, 39]. Recent studies have substantiated the use of these response variables for assessing risk of diabetes [40] and cardiovascular disease [39]. Fiber density was expressed as absolute intake of fiber (g/d) divided by total daily energy intake (MJ). Percentage energy intake from sugars and fat were calculated by dividing energy intake from sugars (kJ) and fat (kJ) respectively by total energy intake (kJ) and then multiplying by 100. SFA:PUFA ratio was expressed as the former divided by the latter.

A total of 48 food groups were created for use as predictors in the RRR analyses (Additional file 3: Table S3) to produce the DPs. Based on the AHS food grouping [27], foods were grouped into the following categories: non-alcoholic beverages (1 group), cereals (7 groups), fats and oils (2 groups), fruit (8 groups), vegetables (8 groups), meat and alternatives (7 groups), dairy (5 groups), soups and sauces (2 groups), snacks and confectionary (4 groups) and alcoholic beverages (3 groups). The number of extracted patterns is dependent on the number of response variables, thus intakes (g/d) of all 48 food groups and the three response variables were used to derive three DPs. These groups were RRR calculated linear functions of food group intakes (dietary patterns) that explained variation in the response variables. A detailed explanation of the RRR methodology can be found elsewhere [12].

To derive a DP score that captured the food groups that contributed most to the RRR pattern, a second, simplified score was derived and used for a sensitivity analysis. This score included food groups with factor loadings greater than > 0.20 [15] and was generated by summing the standardised food group intakes (z-scores) [41]. The simplified score for DP-1 summed positive loadings for pome fruit, wholegrain bread, wholegrain cereals, nuts and seeds, carrots, brassica vegetables, other vegetables, and negative loadings for fruit drinks, high-fat milk, cream, chocolate and non-wholegrain bread. The simplified score for DP-2 summed positive loadings for added sugars, pome fruit, tropical fruit, other fruit, stone fruit and negative loadings for wine and beer and cider. As the third DP explained less than 10% of the variation in response variables it was not further investigated and no sensitivity analysis was conducted.

#### Covariates

Covariates were identified based on previous literature and via a directed acyclic graph (Additional file 4: Figure S1). Socio-demographic characteristics were collected in the NNPAS via interviewer-administered questionnaires. Smoking habits were identified as current, ex-smoker and never smoked. Education status was operationalized as low (completed some high-school or less), medium (completed high-school or completed some high-school and/or certificate/diploma) and high (having a University qualification) [42]. Urban or rural location was defined as major city, inner rural or other [43]. Country of birth was operationalized as born in Australia or another English speaking country (Canada, Ireland, NZ, South Africa, UK, USA) and Other. Information on dieting (currently on a diet to lose weight; currently on a diet for health reasons; currently on a diet to gain weight and not currently on a diet) and atypical dietary intake on day of reporting (much more than usual; usual; much less than usual) were collected. Energy misreporting was calculated as the ratio of energy intake to predicted total energy expenditure (using sex and age-specific equations for a range of weight status, using a physical activity (PA) level of “low active” PA level > =1.4 < 1.6) [44]. PA was assessed according to whether participants met recommendations of 150 min of PA per week and 150 min of PA over 5 or more sessions per week. Time spent sedentary (minutes per day) was defined as sitting or lying down (except when sleeping) for various activities, including time spent sitting at work, and time spent sitting while using computers, watching television, and for other leisure activities. Family history of diabetes was assessed (Yes/No).

#### Statistical analyses

Complete case analysis was used (details of missing data are presented in Fig. 1). Variables were tested for skewness and kurtosis and variables with non-normal distributions, which included BMI, WC, sedentary time and all biomarkers, were logarithmically transformed prior to analysis. Values of logarithmically transformed variables were exponentiated to provide geometric means and SE. DPs identified from RRR were categorized into tertiles. When testing for LDL, TAG and glucose, fasting was required and only fasted data were used. Markers of cardiometabolic health markers were treated as categorical or binary outcome variables for the purpose of descriptive statistics and as continuous variables when evaluating associations with dietary intake. Linear regression analyses were used to test for associations between tertiles of diet quality and DP (independent variables) and markers of cardiometabolic health (dependent variables). Analyses were adjusted in Model 1 for age (continuous), sex, smoking (categorical), physical activity (binary), education (categorical), urban or rural location (categorical), energy misreporting (continuous), dieting (categorical) or atypical dietary intake on day of reporting (categorical) and family history of diabetes (binary). Analyses were further adjusted for BMI (continuous) in Model 2 in order to examine the effect independent of BMI. SAS (version 9; SAS Institute, Cary, NC) was used to derive RRR DPs. Data were analyzed using Stata (version 14; StataCorp., College Station, TX, USA). Survey weightings that were calibrated against population benchmarks (i.e. age, sex and area of usual residence) were used to account for the complex survey design. These weightings were specifically designed by the Australian Bureau of Statistics to account for bias associated with those who volunteered to provide biological samples. *P* < 0.05 was considered statistically significant. No adjustments were made for multiple comparisons given that analyses were pre-defined and methods were comparable with the available literature [17].

## Results

A total of 2121 individuals were included in the present analyses (men: *n* = 960; women: *n* = 1161) (Fig. 1). Characteristics of the omitted sample with the analytical sample were broadly similar, although slightly more adults who were middle aged, highly educated and living in major cities were included in the analytical sample (Additional file 5: Table S4). A total of 31% individuals had high total cholesterol, 22% had low HDL-cholesterol, 31% had high LDL-cholesterol, 13% had high triglycerides, 18% had high apolipoprotein B, 6% had impaired fasting plasma glucose and 7% had HbA1c levels indicative of increased risk of diabetes. Five percent of individuals reported having diabetes, while 29% reported having a family history of diabetes.

### Dietary patterns

The explained variation in food intakes and response variables and the correlations between response variables and DPs are summarized in Table 1. DP-1 was positively correlated with fiber density (*r* = 0.72) and inversely correlated with SFA: PUFA (*r* = − 0.53) and total sugars intake (*r* = − 0.14). DP-2 was positively correlated with fiber density (*r* = 0.31), SFA: PUFA (*r* = 0.24) and total sugars intake (*r* = 0.72). As the third DP explained less than 10% of the variation in response variables it was not further investigated.

**Table 1:** Explained variation (%) in food intakes and response variables for each dietary pattern (DP) as assessed using reduced rank regression and correlation coefficient between DP and response variables for cardiometabolic health outcomes (*n* = 2121)^a^

DP	Explained variation (%)					Correlation coefficient^b,c^		
	Food intakes (total)	Responses (total)	Fibre density (g/MJ)	SFA:PUFA	Sugars (%E)	Fibre density (g/MJ)	SFA: PUFA	Sugars (%E)
DP-1	3.94	26.9	50.9	27.5	2.23	0.72^***^	−0.53^***^	− 0.26^***^
DP-2	2.51	21.0	60.0	31.9	51.8	0.30^***^	0.24^***^	0.72^***^
DP-3	2.47	7.99	65.4	46.0	56.3	0.24^***^	0.36^***^	−0.20^**^

^a^*DP* dietary pattern, *SFA* saturated fatty acid, *PUFA* poly-unsaturated fatty acid, *%E* percentage energy

^b***^Denotes correlation coefficient is significant at *P* < 0.001

^c**^Denotes correlation coefficient is significant at *P* < 0.01

Food groups with the top 5 positive and negative factor loadings for DP-1 and DP-2 are presented in Table 2. A full list of factor loadings for both DPs is presented in Additional file 6: Table S5. DP-1 was characterized by higher intakes of pome fruit, wholegrain bread, wholegrain cereals, nuts and seeds and carrot and root vegetables and lower intakes of fruit drinks, full-fat milk, cream, chocolate and non-wholegrain bread. DP-2 was characterized by higher intakes of added sugars, pome fruit, tropical fruit, other fruit and stone fruit and low intakes of wines, beers and ciders, wholegrain cereals, fish and fried vegetables (Table 2).

**Table 2:** Intakes of response variables and key foods across sex-specific tertiles (T) of dietary pattern (*n* = 2121)^a^

Food groups	Factor loading	Tertile of dietary pattern			*P-*trend^b^
		T1	T2	T3	
DP-1					
Response variables					
Fibre density, g/MJ	–	2.10 ± 0.04	2.75 ± 0.04	3.79 ± 0.06	< 0.001
SFA: PUFA	–	3.55 ± 0.09	2.53 ± 0.06	1.99 ± 0.06	< 0.001
Sugar, %E	–	20.7 ± 0.42	17.7 ± 0.33	18.7 ± 0.37	< 0.001
Direct associations, g/d					
Pome fruit	0.23	51 ± 6	88 ± 11	161 ± 12	< 0.001
Wholegrain bread	0.22	33 ± 5	45 ± 3	82 ± 4	< 0.001
Wholegrain cereals	0.22	31 ± 4	39 ± 3	72 ± 5	< 0.001
Nuts and seeds	0.22	9 ± 1	13 ± 1	29 ± 4	< 0.001
Carrot and root vegetables	0.21	29 ± 3	32 ± 3	72 ± 8	< 0.001
Inverse associations					
Fruit drinks	− 0.24	547 ± 46	283 ± 40	158 ± 17	< 0.001
Full fat milk	− 0.24	447 ± 34	244 ± 20	189 ± 19	< 0.001
Cream	− 0.22	77 ± 8	33 ± 5	20 ± 3	< 0.001
Chocolate	−0.21	23 ± 2	8 ± 1	6 ± 1	< 0.001
Non-wholegrain bread	− 0.20	138 ± 9	108 ± 8	82 ± 6	< 0.001
DP-2					
Response variables					
Fibre density, g/MJ	–	2.52 ± 0.06	2.90 ± 0.05	3.21 ± 0.07	< 0.001
SFA: PUFA	–	2.32 ± 0.07	2.70 ± 0.07	3.07 ± 0.08	< 0.001
Sugar, %E	–	13.9 ± 0.28	18.5 ± 0.37	24.7 ± 0.30	< 0.001
Direct associations, g/d					
Added sugars	0.31	24 ± 2	34 ± 3	53 ± 4	< 0.001
Pome fruit	0.28	52 ± 6	83 ± 8	165 ± 11	< 0.001
Tropical fruit	0.24	45 ± 4	70 ± 6	110 ± 8	< 0.001
Other fruit	0.21	28 ± 3	50 ± 6	77 ± 9	< 0.001
Stone fruit	0.20	18 ± 3	27 ± 4	86 ± 10	< 0.001
Inverse associations, g/d					
Wines	−0.30	281 ± 27	99 ± 13	60 ± 10	< 0.001
Beers and ciders	− 0.30	478 ± 54	187 ± 33	67 ± 13	< 0.001
Non-wholegrain cereals	− 0.19	284 ± 29	200 ± 23	135 ± 13	< 0.001
Fish	− 0.17	70 ± 8	39 ± 5	26 ± 3	< 0.001
Fried vegetables	− 0.15	28 ± 5	21 ± 6	14 ± 3	0.035

^a^*DP* dietary pattern, *SFA* saturated fatty acid, *PUFA* poly-unsaturated fatty acid, *%E* percentage energy; Values represent mean ± SE after adjustment for survey weighting

^b^Linear regression analyses tested for trends across tertiles of dietary pattern. Analyses were adjusted for age and sex

### Diet quality, dietary patterns and demographic characteristics

As shown in Table 3, those individuals with higher DGI were more highly educated, smoked less and were more physically active. Higher DGI scores were associated with higher DP-1 and DP-2 scores, greater fiber density and lower SFA: PUFA ratios. Participants with higher DP-1 scores were older, more physically active, had higher HbA1c levels and higher SBP, while those with higher DP-2 score smoked less and had lower HDL-cholesterol levels (Additional file 7: Table S6).

**Table 3:** Dietary, demographic and cardiometabolic characteristics of Australian adults across sex-specific tertiles (T) of diet quality (*n* = 2121)^a^

Characteristic	All	Diet quality			*P*-trend^b^
		T1	T2	T3	
Dietary					
DGI	81.8 ± 0.58	67.0 ± 0.49	82.1 ± 0.21	96.2 ± 0.41	< 0.001
Dietary pattern 1	0.07 ± 0.04	− 0.58 ± 0.06	0.07 ± 0.07	0.72 ± 0.06	< 0.001
Dietary pattern 2	0.01 ± 0.04	− 0.31 ± 0.06	0.10 ± 0.05	0.24 ± 0.06	< 0.001
Fibre density, g/MJ	2.87 ± 0.04	2.23 ± 0.04	2.85 ± 0.06	3.54 ± 0.07	< 0.001
SFA: PUFA	2.69 ± 0.05	2.89 ± 0.09	2.72 ± 0.08	2.47 ± 0.07	< 0.001
Sugar, %E	19.0 ± 0.24	18.2 ± 0.40	19.5 ± 0.37	19.4 ± 0.39	0.054
Demographic					
Age, y	46.4 ± 0.48	44.3 ± 1.20	47.1 ± 0.95	47.7 ± 1.29	0.11
Female, %	49.8	47.4	48.5	53.6	0.27
Country of birth (English speaking)	79.8	82.2	78.4	78.9	0.51
Highest level of education					
Low	19.5	22.9	18.5	17.1	0.025
Medium	49.6	51.6	52.0	45.2	
High	30.9	25.5	29.6	37.7	
Smoking, %					
Current smoker	11.9	18.1	11.9	5.81	< 0.001
Former smoker	33.4	37.1	32.6	30.3	
Never smoked	54.7	44.8	55.5	63.9	
Meet PA recommendations, %	50.2	42.8	53.5	54.3	0.036
Sedentary time, min/d	343 ± 5.8	350 ± 10.2	336 ± 11.2	344 ± 8.43	0.64
Cardiometabolic					
HbA1c, mmol/mol	35.6 ± 0.20	35.6 ± 0.32	35.9 ± 0.30	35.4 ± 0.36	0.71
Plasma glucose, mmol/L	5.08 ± 0.02	5.12 ± 0.04	5.12 ± 0.04	5.00 ± 0.04	0.050
Total cholesterol, mmol/L	5.00 ± 0.04	5.00 ± 0.05	5.00 ± 0.07	5.00 ± 0.05	0.88
HDL cholesterol, mmol/L	1.35 ± 0.12	1.35 ± 0.02	1.36 ± 0.02	1.34 ± 0.02	0.57
LDL cholesterol, mmol/L	3.08 ± 0.03	3.07 ± 0.05	3.08 ± 0.06	3.08 ± 0.04	0.72
Triglycerides, mmol/L	1.23 ± 0.02	1.23 ± 0.04	1.20 ± 0.04	1.27 ± 0.04	0.76
Apolipoprotein B, g/L	1.00 ± 0.01	1.00 ± 0.18	1.00 ± 0.02	1.01 ± 0.01	0.29
BMI, kg/m^2^	27.0 ± 0.19	27.4 ± 0.34	26.7 ± 0.32	26.8 ± 0.35	0.23
BMI category, %					
Underweight/normal weight	40.4	38.1	39.4	43.7	0.32
Overweight	35.1	33.7	36.4	35.1	
Obese	24.5	28.2	24.2	21.2	
Waist circumference, cm	91.3 ± 0.46	92.6 ± 0.91	91.0 ± 0.90	90.3 ± 0.94	0.14
Systolic blood pressure, mmHg	121 ± 0.58	120 ± 1.11	121 ± 1.10	122 ± 1.18	0.36
Diastolic blood pressure, mmHg	75.6 ± 0.39	75.4 ± 0.75	75.4 ± 0.62	76.0 ± 0.84	0.65
Overall cardiometabolic risk score	− 0.11 ± 0.02	− 0.10 ± 0.04	− 0.11 ± 0.04	− 0.12 ± 0.04	0.69

^a^*BMI* body mass index, *DP* dietary pattern; Education: low (completed some high-school or less), medium (completed high-school or completed some high-school and/or certificate/diploma) and high (having a tertiary qualification). BMI category: underweight/normal weight (BMI < 25 kg/m^2^), overweight (25 ≤ BMI < 30 kg/m^2^), obese (BMI ≥ 30 kg/m^2^); Overall cardiometabolic risk score was based on WC, TAG, HDL-cholesterol, blood pressure (average blood pressure was used as an index for systolic and diastolic blood pressure), and fasting plasma glucose based on an established methodology [30]. Values represent mean ± SE after adjustment for survey weighting. Where transformed for regression analyses, values represent exponentiated geometric mean ± SE

^b^Linear regression analyses (continuous variables) and χ^2^ (categorical variables) were used to test for trends across tertiles

### Diet quality, dietary patterns and cardiometabolic health

Higher DGI was associated with lower plasma glucose levels, BMI and WC. No other significant associations between DGI and cardiometabolic markers were observed. Following adjustment for BMI, the relationship between DGI and plasma glucose was attenuated but remained significant (Table 4).

**Table 4:** Multivariable adjusted regression coefficients for cardiometabolic risk markers per sex-specific tertiles (T) of dietary guideline index (DGI) (*n* = 2121)^a^

Characteristic	Tertile of DGI			*P-*trend^b^
	T1	T2	T3	
HbA1c (mmol/mol)				
Model 1	ref	0.001 (0.008)	− 0.004 (0.010)	0.70
Model 2	ref	0.004 (0.010)	− 0.001 (0.010)	0.90
Plasma glucose (mmol/L)				
Model 1	ref	− 0.004 (0.009)	− 0.024 (0.009)	0.008
Model 2	ref	0.001 (0.009)	−0.019 (0.009)	0.033
Total cholesterol (mmol/L)				
Model 1	ref	−0.001 (0.016)	−0.004 (0.017)	0.82
Model 2	ref	0.004 (0.016)	−0.003 (0.017)	0.98
HDL cholesterol (mmol/L)				
Model 1	ref	−0.003 (0.021)	−0.029 (0.023)	0.20
Model 2	ref	−0.014 (0.020)	−0.041 (0.023)	0.08
LDL cholesterol (mmol/L)				
Model 1	ref	0.008 (0.023)	0.002 (0.025)	0.93
Model 2	ref	0.014 (0.022)	0.009 (0.025)	0.73
Triglycerides (mmol/L)				
Model 1	ref	−0.020 (0.043)	0.025 (0.033)	0.44
Model 2	ref	0.002 (0.041)	0.047 (0.034)	0.17
Apolipoprotein B (g/L)				
Model 1	ref	0.021 (0.024)	0.033 (0.025)	0.20
Model 2	ref	0.030 (0.023)	0.043 (0.025)	0.10
BMI (kg/m^2^)				
Model 1	ref	−0.033 (0.015)	−0.033 (0.014)	0.019
Model 2	–	–	–	–
Waist circumference (cm)				
Model 1	ref	−0.022 (0.013)	−0.028 (0.010)	0.008
Model 2	–	–	–	–
Systolic blood pressure (mmHg)				
Model 1	ref	−0.642 (1.153)	0.329 (1.591)	0.83
Model 2	ref	−0.164 (1.120)	0.805 (1.591)	0.61
Diastolic blood pressure (mmHg)				
Model 1	ref	−0.494 (0.834)	−0.076 (1.161)	0.95
Model 2	ref	0.078 (0.765)	0.495 (1.160)	0.67
Overall cardiometabolic risk score				
Model 1	ref	−0.044 (0.050)	−0.030 (0.041)	0.48
Model 2	–	–	–	–

^a^*BMI* body mass index, Values represent regression coefficients and SE. Overall cardiometabolic risk score was based on WC, TAG, HDL-cholesterol, blood pressure (average blood pressure was used as an index for systolic and diastolic blood pressure), and fasting plasma glucose based on an established methodology [30]

^b^Linear regression analyses were used to test for significant differences across tertiles of diet quality score. Analyses were adjusted for Model 1 and Model 2. Model 1 adjusted for age (continuous), sex, smoking (categorical), physical activity (binary), education (categorical), urban or rural location (categorical), energy misreporting (continuous), dieting (categorical) or atypical dietary intake on day of reporting (categorical) and family history of diabetes. Blood biomarkers and blood pressure outcomes were further adjusted for BMI in Model 2

Higher DP-1 was associated with lower BMI and WC (Table 5). Higher DP-2 was associated with lower HDL-C. There was a trend towards lower DBP. Following adjustment for BMI, the relationship between DP-2 and HDL-cholesterol was attenuated but remained significant and the association with DBP became significant (Table 6). No associations between DPs and other markers were observed.

**Table 5:** Multivariable adjusted regression coefficients for cardiometabolic risk markers per sex-specific tertiles (T) of dietary pattern 1 (*n* = 2121)^a^

Characteristic	Tertile of dietary pattern			*P-*trend^b^
	T1	T2	T3	
HbA1c (mmol/mol)				
Model 1	ref	0.008 (0.007)	0.004 (0.001)	0.26
Model 2	ref	0.007 (0.010)	0.020 (0.013)	0.13
Plasma glucose (mmol/L)				
Model 1	ref	−0.017 (0.001)	−0.007 (0.010)	0.52
Model 2	ref	−0.010 (0.008)	0.002 (0.010)	0.84
Total cholesterol (mmol/L)				
Model 1	ref	−0.020 (0.016)	−0.022 (0.019)	0.26
Model 2	ref	−0.015 (0.016)	−0.017 (0.019)	0.40
HDL cholesterol (mmol/L)				
Model 1	ref	0.032 (0.021)	0.003 (0.020)	0.93
Model 2	ref	0.017 (0.022)	−0.016 (0.019)	0.37
LDL cholesterol (mmol/L)				
Model 1	ref	−0.035 (0.023)	−0.027 (0.029)	0.37
Model 2	ref	−0.027 (0.024)	−0.016 (0.028)	0.58
Triglycerides (mmol/L)				
Model 1	ref	−0.063 (0.032)	−0.067 (0.036)	0.07
Model 2	ref	−0.031 (0.033)	−0.030 (0.035)	0.41
Apolipoprotein B (g/L)				
Model 1	ref	−0.041 (0.025)	−0.022 (0.028)	0.46
Model 2	ref	−0.027 (0.025)	−0.005 (0.026)	0.88
BMI (kg/m^2^)				
Model 1	ref	−0.048 (0.012)	− 0.056 (0.015)	< 0.001
Model 2	–	–	–	–
Waist circumference (cm)				
Model 1	ref	−0.032 (0.009)	−0.035 (0.010)	0.001
Model 2	–	–	–	–
Systolic blood pressure (mmHg)				
Model 1	ref	1.431 (1.132)	0.753 (1.376)	0.61
Model 2	ref	2.132 (1.091)	1.571 (1.286)	0.25
Diastolic blood pressure (mmHg)				
Model 1	ref	0.708 (0.879)	−0.566 (1.024)	0.56
Model 2	ref	1.530 (0.850)	0.394 (1.004)	0.74
Overall cardiometabolic risk score				
Model 1	ref	−0.097 (0.038)	−0.082 (0.042)	0.061
Model 2	–	–	–	–

^a^*BMI* body mass index; Values represent regression coefficients and SE. Overall cardiometabolic risk score was based on WC, TAG, HDL-cholesterol, blood pressure (average blood pressure was used as an index for systolic and diastolic blood pressure), and fasting plasma glucose based on an established methodology [30]

^b^Linear regression analyses were used to test for significant differences across tertiles of dietary pattern score. Analyses were adjusted for Model 1 and Model 2. Model 1 adjusted for age (continuous), sex, smoking (categorical), physical activity (binary), education (categorical), urban or rural location (categorical), energy misreporting (continuous), dieting (categorical) or atypical dietary intake on day of reporting (categorical) and family history of diabetes. Blood biomarkers and blood pressure outcomes were further adjusted for BMI in Model 2

**Table 6:** Multivariable adjusted regression coefficients for cardiometabolic risk markers per sex-specific tertiles (T) of dietary pattern 2 (*n* = 2121)^a^

Characteristic	Tertile of dietary pattern			*P-*trend^b^
	T1	T2	T3	
HbA1c (mmol/mol)				
Model 1	ref	−0.003 (0.010)	0.008 (0.009)	0.37
Model 2	ref	−0.004 (0.009)	0.007 (0.009)	0.44
Plasma glucose (mmol/L)				
Model 1	ref	−0.009 (0.008)	−0.007 (0.008)	0.35
Model 2	ref	−0.019 (0.008)	−0.009 (0.007)	0.24
Total cholesterol (mmol/L)				
Model 1	ref	−0.033 (0.018)	−0.009 (0.017)	0.62
Model 2	ref	−0.033 (0.018)	−0.010 (0.017)	0.57
HDL cholesterol (mmol/L)				
Model 1	ref	−0.035 (0.022)	−0.056 (0.024)	0.022
Model 2	ref	−0.034 (0.021)	−0.053 (0.023)	0.028
LDL cholesterol (mmol/L)				
Model 1	ref	−0.023 (0.027)	0.014 (0.026)	0.57
Model 2	ref	−0.024 (0.026)	0.012 (0.025)	0.61
Triglycerides (mmol/L)				
Model 1	ref	−0.063 (0.041)	−0.018 (0.040)	0.69
Model 2	ref	−0.064 (0.042)	−0.024 (0.040)	0.56
Apolipoprotein B (g/L)				
Model 1	ref	−0.026 (0.026)	0.013 (0.025)	0.57
Model 2	ref	−0.026 (0.025)	0.010 (0.023)	0.64
BMI (kg/m^2^)				
Model 1	ref	0.001 (0.016)	0.010 (0.014)	0.49
Model 2	–	–	–	–
Waist circumference (cm)				
Model 1	ref	−0.003 (0.012)	0.002 (0.010)	0.84
Model 2	–	–	–	–
Systolic blood pressure (mmHg)				
Model 1	ref	1.885 (1.286)	−0.435 (1.090)	0.67
Model 2	ref	1.871 (1.298)	−0.573 (1.051)	0.57
Diastolic blood pressure (mmHg)				
Model 1	ref	0.275 (0.871)	−1.811 (0.969)	0.07
Model 2	ref	0.258 (0.818)	−1.979 (0.908)	0.033
Overall cardiometabolic risk score				
Model 1	ref	0.002 (0.044)	0.010 (0.046)	0.83
Model 2	–	–	–	–

^a^BMI, body mass index; Values represent regression coefficients and SE. Overall cardiometabolic risk score was based on WC, TAG, HDL-cholesterol, blood pressure (average blood pressure was used as an index for systolic and diastolic blood pressure), and fasting plasma glucose based on an established methodology [30]

^b^Linear regression analyses were used to test for significant differences across tertiles of dietary pattern score. Analyses were adjusted for Model 1 and Model 2. Model 1 adjusted for age (continuous), sex, smoking (categorical), physical activity (binary), education (categorical), urban or rural location (categorical), energy misreporting (continuous), dieting (categorical) or atypical dietary intake on day of reporting (categorical) and family history of diabetes. Blood biomarkers and blood pressure outcomes were further adjusted for BMI in Model 2

### Sensitivity analyses

Patterns of significant results remained consistent when associations between simplified DP scores and cardiometabolic health were investigated. Simplified DP-1 was associated with lower BMI (coef − 0.023, SE 0.009; P-trend = 0.012) and WC (coef − 0.014, SE 0.006; P-trend = 0.023) following adjustment for Model 1. Simplified DP-2 was associated with lower HDL-cholesterol (coef − 0.033, SE 0.010; P-trend = 0.001) and DBP (coef − 1.277, SE 0.418; P-trend = 0.003) following adjustment for Model 2.

## Discussion

The aim of this study was to investigate the relationship between DPs, diet quality and cardiometabolic health in a nationally-representative sample of Australian adults. Our main findings are that both higher diet quality and a healthier DP (DP1) were primarily associated with favorable anthropometric markers of cardiometabolic health (BMI, WC) independent of numerous potential demographic and health-related confounders. Although effect sizes were small, these findings highlight the consistency of a diet quality and DP methodology to estimate associations with anthropometric markers of cardiometabolic health. Previous studies have independently compared diet quality scores [17] and DP methodologies [20] with markers of cardiometabolic health. However, few studies simultaneously compare multiple measures of overall diet with cardiometabolic health.

Evidence for an association between diet quality and markers of cardiometabolic health is mixed. Consistent with our findings, in a sample of Hispanic adults, higher 2010 Alternative HEI was associated with lower WC and glucose levels [18]. Moreover, studies in Australian adult populations have shown that higher DGI was associated with lower glucose levels [19, 45]. However, given the strength of the association between DGI and glucose levels observed in our study, we cannot discount the possibility of this being a chance finding. In line with a recent cross-sectional study of multiple diet quality scores in US women (predominantly Caucasian) [17], but in contrast with findings for the 2005 HEI (50% Caucasian) [46], diet quality was not associated with total or HDL-cholesterol. Similar inconsistencies are evident for other markers, such as TAG and HbA1c [46]. The inconsistency of associations with markers of cardiometabolic health may be partly attributable to differences in the ethnicity of the sample population [47], given that 80% of our sample were born in Australia or another English speaking country. However, it is likely to largely be due to methodological differences, such as reverse causation given the cross-sectional design, the method of assessing diet quality, choice of covariates and variation in sample sizes and resulting statistical power. To mitigate individual differences in markers, we evaluated an overall cardiometabolic risk score. This score has been used primarily for dietary behaviours [48] rather than patterns and so warrants further investigation.

The role of DPs in relation to cardiometabolic health is mixed, with only a small number of studies utilizing RRR [13, 49–51]. Of these studies, some have used biochemical response variables, such as cholesterol, to derive associations between DPs and markers of cardiovascular health [25] and risk [13]. Associations observed between DP-1, consistent with a ‘healthy’ DP, and anthropometric outcomes are comparable to other studies [20, 25, 52–54]. In a recent analysis of 10,008 individuals from the Multiethnic Cohort, a RRR-derived DP (using four biomarkers as response variables) and low in fruit drinks and white rice and high in whole grains and fruits was inversely associated with BMI [13]. Few studies have use nutrient intakes as response variables. In a longitudinal study of 2037 Swedish adults, an ‘unhealthy’ RRR-derived DP characterized by some similar response variables to our study (high dietary energy density, SFA and low fiber-density) was associated with greater adiposity, cholesterol, TAG, SBP and DBP but not with CVD endpoints [52]. However, comparability of results was limited as this study was conducted in obese individuals only, who may be more metabolically sensitive to an ‘unhealthy’ diet [55]. Recent data from a AHS study showed that a ‘healthy’ DP, derived using factor analysis and characterized by high intakes of wholegrains and fresh fruit and low intakes of take-away foods and soft drinks, was associated with higher odds of having a healthy metabolic profile [20]. Similar findings for a ‘healthy’ DP were observed in a national longitudinal study in Chinese adults [53] and a representative community sample of Lebanese adults [54] but were mixed in a prospective study of UK males [56]. Our DPs were derived to explain the maximum variation in dietary energy density, SFA: PUFA, and total sugars; it is likely that a DP that explained other nutrient intakes, as well as a DP derived using other methodologies, may show different associations with cardiometabolic health.

Observed associations between DP-2 and cardiometabolic health outcomes requires further investigation. Positive correlations with all response variables resulted in DP-2 food groups that were less consistent with a ‘healthy’ or ‘unhealthy’ diet, i.e. DP-2 was high in both added sugar and fruit intake and low in fish, alcohol and non-whole grain cereals. Although other studies have used a ratio of SFA:PUFA [15] and total sugars, [14] the use of a ratio and a lack of specificity of sugar type (added vs natural) may have limited the interpretation of DP-2. Moreover, this may partly explain why we observed inverse associations with both HDL-cholesterol and DBP. Given that RRR derives DPs that best represent the chosen response variables, investigation of alternative response variables is needed to better understand the role of RRR-derived DPs in cardiometabolic heath. In addition, limited RRR research has examined the role of certain individual biomarkers, such as apolipoprotein, which has been linked to better cardiometabolic health using other DP methodologies [57].

Our findings show consistency between two whole-diet based methodologies for identifying associations with anthropometric markers of cardiometabolic health. Foods comprise a complex mixture of nutrients with potentially contrasting associations with cardiometabolic health, thus supporting a whole diet approach. [58] Future studies based on RRR should evaluate the mechanistic role of response variables and the use of comparable methodologies for deriving an overall risk score.

### Strengths and limitations

The present study has a number of strengths. This study was conducted in a large, nationally representative survey of Australian adults. Although the generalizability of our sample may have been limited by non-response bias associated with those who volunteered to provide biological samples, our analyses used survey weightings that were specifically designed to account for such bias. Moreover, there was minimal difference in characteristics between the omitted sample and the analytical sample (Additional file 5: Table S4). We derived two whole diet methodologies, which facilitated a comparison between data-driven and dietary adherence-based methodologies within the same population. These scores were derived from two 24-h recalls, thus offering a more accurate estimate of dietary intake than FFQ-based scores [59]. Moreover, the DGI used age and sex-specific cut-offs, thereby increasing the accuracy of diet quality scores across different population groups. A further strength of this study is the evaluation of the role of BMI in the association between diet quality, DPs and cardiometabolic health.

A limitation of this study is its cross-sectional design, which prohibited interpretation of causal relationships. Given that some measures of cardiometabolic health were self-reported, some individuals may have been aware of their poor cardiometabolic health and may have changed their diet as a result. Thus, we cannot discount the possibility of reverse causality. Information on blood pressure or lipid lowering medication was not available and so we were unable to exclude or adjust for this potential confounder. Thus, we cannot discount bias associated with incongruences between blood pressure and lipid data before and after anti-hypertensive and lipid lowering medication use and any associated behavioral changes. While our analyses were adjusted for multiple confounders, including energy misreporting, residual confounding may be a limitation. Moreover, missing data, most notably for biochemical and the second day of dietary recalls, may have introduced bias. Although the timing of 24-h dietary recalls may limit their ability to capture usual intake and seasonal variations in dietary intakes, our use of two 24-h recalls offers an advantage over previous studies based on one day of dietary recall [20] and our research has demonstrated that RRR DPs derived from the average of two days are comparable to those derived using usual intakes [14]. Any seasonal impact on DPs may have influenced smaller food groups, such as stone fruits, but is likely to be minimal in larger food groups, such as brassica vegetables, and in the DGI. Moreover, a seasonal adjustment was also incorporated into the person weights in the NNPAS [27]. Limitations of RRR should also be acknowledged. First, although the food groups are based on AUSNUT 2011–13, the number and definitions of the food groups used in this study may have affected the derived DP. Second, although our choice of response variables was based on published literature the use of different response variables may have resulted in a different DPs. Moreover, RRR derives DPs that closely reflect nutrient intakes or intermediate markers, which may result in DPs less consistent with behavioral patterns compared to factor or cluster analysis. Third, we generated and fitted RRR DP in the same data set. To rule out any effect of over-fitting and to show generalizability of RRR DP, future studies should consider deriving and applying DP in independent data sets. Although we were unable to disaggregate the effect of added sugars from total sugars in our response variable, food group intakes provided information on the foods that characterized the DPs. Prospective studies that consider type 2 diabetes and cardiovascular disease incidence and that compare both diet quality and DP methodologies within the same population are needed.

### Implications of findings

Both diet quality and DP methodologies (DP-1) support healthy eating initiates to improve cardiometabolic health that centre on diets rich in fruits, vegetables, wholegrains and lean meats and/or alternatives and low in processed foods and alcohol. Furthermore, this study provides evidence for the comparability of associations between whole diet measures and cardiometabolic health. This evidence is imperative for the effective integration of diet quality and DP research. As a result, the present findings have the potential to inform the design of future DP-based research that aims to evaluate association with cardiometabolic health.

## Conclusions

Better diet quality and a healthier DP were primarily associated with favorable anthropometric markers of cardiometabolic health. Findings support the need to compare across multiple whole-diet based methodologies, which take into consideration the interaction between foods and nutrients consumed together. The limited associations observed between diet quality, DPs and biochemical cardiometabolic markers warrants further investigation. Studies that evaluate the role of different response variables and that are longitudinal in design are needed to better understand causal relationships between diet and cardiometabolic health.

## Additional files

Additional file 1: Table S1. STROBE-nut: An extension of the STROBE statement for nutritional epidemiology. (DOCX 22 kb)

Additional file 2: Table S2. Components and scoring methods of the Dietary Guideline Index (DGI). (DOCX 18 kb)

Additional file 3: Table S3. Food groups used as predictors in the reduced rank regression analyses (*n* = 48) (DOCX 17 kb)

Additional file 4: Figure S1. Directed acyclic graph (DAG) used to help identify the confounder selection for the statistical analysis. (DOCX 189 kb)

Additional file 5: Table S4. Characteristics of adults who were omitted from the analytical sample based on only 1 day of 24-h recall and based on missing covariates and those who were included in the analytical sample. (DOCX 15 kb)

Additional file 6: Table S5. Factor loadings for reduced rank regression dietary patterns. (DOCX 19 kb)

Additional file 7: Table S6. Demographic and cardiometabolic characteristics of Australian adults across sex-specific tertiles (T) of each dietary pattern (*n* = 2121). (DOCX 21 kb)

## Abbreviations

AHS: Australian Health Survey
BMI: Body mass index
DBP: Diastolic blood pressure
DGI: Dietary guideline index
DP: Dietary pattern
FFQ: Food frequency questionnaire
NHMS: National Health Measures Survey
NNPAS: National Nutrition and Physical Activity Survey
PA: Physical activity
SBP: Systolic blood pressure
