# Supplementary material for: Association between diet quality, dietary patterns and cardiometabolic health in Australian adults: a cross-sectional study
Source: Nutr J. 2018 Feb 12;17:19. doi: 10.1186/s12937-018-0326-1 (PMC5809905; doi:10.1186/s12937-018-0326-1)
Supplement: Supplementary file 2 — Components and scoring methods of the Dietary Guideline Index (DGI). (DOCX 18 kb) [file 12937_2018_326_MOESM2_ESM.docx]

**Table S2** Components and scoring methods of the Dietary Guideline Index (DGI)

| Dietary Guideline | Indicator and description | Criteria for maximum score^1^ | Criteria for minimum score (0) | Maximum Score |
| --- | --- | --- | --- | --- |
| Guidelines for adequate intake | |  |  |  |
| 1. Enjoy a wide variety of nutritious foods | Food variety ^2^: proportion of food from each of the 5 core food groups eaten at least one serve per week | 100% | 0% | 10 |
| 2. Plenty of vegetables | Total vegetable intake: servings of vegetables per day | 19-50 y: M ≥ 6, F ≥ 5 | 0 | 10 |
|  |  | 51-70 y: M ≥ 5.5, F ≥ 5 |  |  |
|  |  | >70 y: M ≥ 5, F ≥ 5 |  |  |
| 3. Fruit | Total fruit intake: servings of fruit per day | ≥ 2 | 0 | 10 |
| 4. Grain (cereal) foods | Total cereal intake: servings of grains per day | 19-50 y: M ≥ 6, F ≥ 6 | 0 | 5 |
|  |  | 51-70 y: M ≥ 6, F ≥ 4 |  |  |
|  |  | >70 y: M ≥ 4.5, F ≥ 3 |  |  |
|  | Wholegrain or high fibre cereals: proportion of wholegrain bread to white bread intake per day | ≥ 50% wholemeal bread | 0% | 5 |
| 5. Lean meat and poultry, fish, eggs, nuts and seeds, and legumes/beans | Total lean meat and alternative: servings per day | 19-50 y: M ≥ 3, F ≥ 2.5 | 0 | 5 |
|  |  | 51-70 y: M ≥ 2.5, F ≥ 2 |  |  |
|  |  | >70 y: M ≥ 2.5, F ≥ 2 |  |  |
|  | Lean meat: proportion of lean meats and alternatives to total meat and alternatives per day | 100% | 0% | 5 |
| 6. Milk, yoghurt, cheese and/or their alternatives^3^ | Total dairy and alternative: servings per day | 19-50 y: M ≥ 2.5, F ≥ 2.5 | 0 | 10 |
|  |  | 51-70 y: M ≥ 2.5, F ≥ 4 |  |  |
|  |  | >70 y: M ≥ 3.5, F ≥ 4 |  |  |
| 7. Drink plenty of water | Total beverage intake^4^: servings per day | M ≥ 10; F ≥ 8 | 0 | 5 |
|  | Water^5^: proportion of water to total beverage intake per day | ≥ 50% | 0% | 5 |
| Guidelines to limit or moderate intake | |  |  |  |
| 8. Limit intake of foods containing saturated fat, added salt, added sugars and alcohol | Limit discretionary foods | M ≤ 3; F ≤ 2.5 | M > 3; F > 2.5 | 10 |
| 9. Limit intake of foods high in saturated fat | Trim meat: proportion of trimmed meat to total meat | ≥ 50% | 0% | 5 |
|  | Choose reduced-fat milk: proportion of reduced-fat milk to total milk intake per day | ≥ 50% reduced-fat milk | 0% | 5 |
| 10. Small allowance of unsaturated oils, fats or spreads | Unsaturated spreads and oils: servings per day | 19-50 y: M ≤ 4, F ≤ 2 | M > 4; F > 2 | 10 |
|  |  | 51-70 y: M ≤ 4, F ≤ 2 |  |  |
|  |  | >70 y: M ≤ 2, F ≤ 2 |  |  |
| 11. Limit intake of foods and drinks containing added salt | Salt use: salt added during cooking | Never or rarely | Usually | 5 |
|  | Salt use: salt added during the meal | Never or rarely | Usually | 5 |
| 12. Limit intake of foods and drinks containing added sugars | Limit extra sugar^6^: servings per day | M ≤ 1.5; F ≤ 1.25 | M > 1.5; F > 1.25 | 10 |
| 13. If you choose to drink alcohol, limit intake | Limit alcohol: servings per day | ≤ 2 | > 2 | 10 |

1. Criteria for maximum scores were derived from the Australian Dietary Guidelines unless otherwise noted.

2. Food variety was measured and scored using a similar method to the Recommended Food Score.

3. Choosing reduced fat dairy is captured in the “Limit intake of foods high in saturated fat” component.

4. The Eat for Health Australian Dietary Guidelines do not have specific recommendations for beverage consumption and recommended the guidelines found in the Nutrient Reference Values for Australia and New Zealand (30).

5. The proportion of water to total beverage intake was derived from US beverage guidelines.

6. Since added sugar intake is not recommended there are no cut-off values for number or recommended serves, instead half of the maximum discretionary food cut-off were used consistent with the original DGI.
