# Supplementary material for: Association between diet quality, dietary patterns and cardiometabolic health in Australian adults: a cross-sectional study
Source: Nutr J. 2018 Feb 12;17:19. doi: 10.1186/s12937-018-0326-1 (PMC5809905; doi:10.1186/s12937-018-0326-1)
Supplement: Supplementary file 3 — Food groups used as predictors in the reduced rank regression analyses (n = 48) (DOCX 17 kb) [file 12937_2018_326_MOESM3_ESM.docx]

**Table S3** Food groups used as predictors in the reduced rank regression analyses (n=48)

| **Categories** | **Food groups** |
| --- | --- |
| Non-alcoholic beverages (1 food group) | 1. Tea, coffee, sports drinks, other beverages excluding fruit juices |
| Cereals (7 food group) | 2. Dark, wholegrain, mixed grain breads, bread rolls, such as whole wheat, rye, or pumpernickel  3. High fibre cereals (bran, granola, or shredded wheat, museli; porridge) and wholegrain pasta and brown rice |
|  | 4. Non-wholegrain bread/bread rolls (incl fried toast)  5. Non-wholegrain breakfast cereal and rice/pasta (including filed); quinoa; couscous; noodles  6. Dry or savoury biscuits, crisp bread, crackers  7. Processed cakes and pastries (cakes, sweet muffins, scones or pikelets; sweet pies or sweet pastries; plain sweet biscuits; cream, chocolate biscuits; Other puddings or desserts  8. Pies, sausage roll or other savoury pastries; pizza |
| Fats and oils (2 food group) | 9. Unsaturated margarine and oils |
|  | 10. Butter, animal-based solid fats and high fat dairy blends |
| Fruit (8 food group) | 11. Pomme fruit (apples, pears, other pomme fruit)  12. Berry fruit (blackberry, blueberry, cranberry, mulberry, raspberry and strawberry)  13. Citrus fruit (oranges, grapfruit,lemon, lime, cumquat, mandarin, tangelo, tangerine and other citrus fruit)  14. Stone fruit (nectarine, peach, apricot, plum, cherry and other stone fruit);  15. Tropical and subtropical fruit (banana, pinapple, babaco, cheese fruit, fig, persimmon, tamarillo, wax jambo, custard apple, feijoa, guava, jackfruit, lychee, mango, passionfruit, pawpaw, pomegranate, prickly pear, rambutan, tropical fruit)  16. Other fruit (eg. grape, kiwifruit, melon, pepino, rhubarb, quandong, dried fruit)  17. Fruit juice (including smoothie) |
|  | 18. Fruit drinks, cordials and soft drinks |
| Vegetables (8 food group) | 19. Green (lettuce, spinach, asparagus etc) and brassica vegetables (cabbage, cauliflower and similar brassica vegetables)  20. Legumes (beans, chickpea, lentil, split pea, tofu, soybean curd, tempeh)  21. Carrot and root vegetables (carrot, swede, beetroot etc)  22. Starchy vegetables (potato, sweet potato, sweetcorn)  23. Tomato and tomato products  24. Peas and beans  25. Other vegetables (mushroom, pumpkin, capsicum etc) |
|  | 26. Fried vegetables (i.e. potato fries) |
| Meats and alternatives (7 food group) | 27. Chicken, turkey, (semi-trimmed, fully-trimmed, separable lean, lean, no skin)  28. Fish (raw, baked, roast, fried, grilled or BBQ’d)  29. Eggs  30. Nuts and seeds (peanuts, peanut butter, other nut spreads, other nuts (e.g. almonds, walnuts), seeds (e.g. sunflower, tahini)  31. Other alternatives: soybeans or tofu, baked beans, other beans (e.g. chichpeas)/lentils) |
|  | 32. Trimmed meat: Beef, veal, kangaroo, rabbit, venison, goat (<5% fat, 5-10% fat, semi-trimmed, fully-trimmed, separable lean); Lamb (semi-trimmed, fully-trimmed, separable lean); Pork (semi-trimmed, fully-trimmed, separable lean)  33. Untrimmed meat: Beef, veal, kangaroo, rabbit, venison, goat, lamb, pork;  34. Processed/battered meat |
| Dairy (5 food group) | 35. 2%, 1% or skim milk (including soy) and beverages (including flavoured milk drinks)  36. Reduced, skim or no fat natural or flavoured yoghurt and reduced fat cheese |
|  | 37. Regular fat milk (including soy) and beverages (including flavoured milk drinks)  38. Regular fat natural or flavoured yoghurt (e.g. 2 or 3%) and reduced fat cheese  39. Cream or sour cream; Ice cream; custard; other dairy desserts |
| Soups, sauces, dips (2 food group) | 40. Soups: homemade, dry mix, canned  41. Sauces, dips and high-fat dressings |
| Snacks and confectionary (4 food group) | 42. Snack foods: potato snacks, corn snacks  43. Sugar products and dishes  44. Chocolate  45. Other confectionary |
| Alcoholic beverages (3 food group) | 46. Beers and ciders  47. Wines  48. Spirits and other alcoholic beverages |
