# Supplementary material for: Association between diet quality, dietary patterns and cardiometabolic health in Australian adults: a cross-sectional study
Source: Nutr J. 2018 Feb 12;17:19. doi: 10.1186/s12937-018-0326-1 (PMC5809905; doi:10.1186/s12937-018-0326-1)
Supplement: Supplementary file 4 — Directed acyclic graph (DAG) used to help identify the confounder selection for the statistical analysis. (DOCX 189 kb) [file 12937_2018_326_MOESM4_ESM.docx]

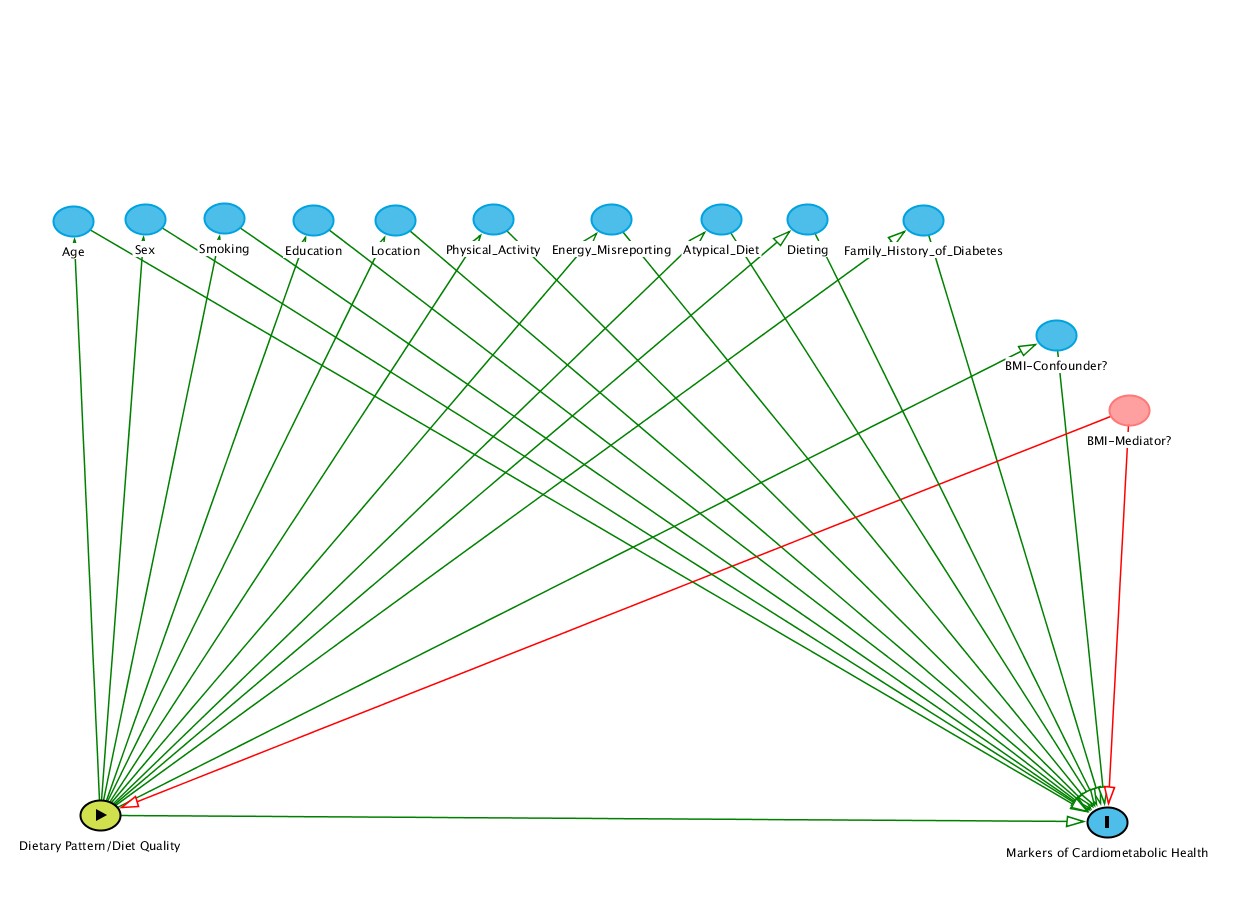


**Figure S1** Directed acyclic graph (DAG) used to help identify the confounder selection for the statistical analysis. This DAG resulted in inclusion of 2 models in the analysis of the association of dietary patterns, diet quality and markers of cardiometabolic health: model 1: age (continuous) sex, smoking (categorical), education (categorical), location (categorical), physical activity (binary), energy misreporting (continuous), dieting (categorical) or atypical dietary intake on day of reporting (categorical) and family history of diabetes (binary) and model 2: model 1 plus BMI (continuous) for associations with blood pressure and biomarkers. Green lines represent a confounder. Red lines represent a mediator.
