# Supplementary material for: Association between diet quality, dietary patterns and cardiometabolic health in Australian adults: a cross-sectional study
Source: Nutr J. 2018 Feb 12;17:19. doi: 10.1186/s12937-018-0326-1 (PMC5809905; doi:10.1186/s12937-018-0326-1)
Supplement: Supplementary file 5 — Characteristics of adults who were omitted from the analytical sample based on only 1 day of 24-h recall and based on missing covariates and those who were included in the analytical sample. (DOCX 15 kb) [file 12937_2018_326_MOESM5_ESM.docx]

**Table S4** Characteristics of adults who were omitted from the analytical sample based on only 1 day of 24-hour recall and based on missing covariates and those who were included in the analytical sample

| Characteristic | Sub-category | Omitted sample based on only 1 x 24 hour recall (n=3288) | Omitted sample based on other missing data (n=3784) | Analytical sample  (n=2121) |
| --- | --- | --- | --- | --- |
| Sex | Male | 1520 (51.6) | 1802 (49.2) | 960 (50.2) |
|  | Female | 1768 (48.4) | 1982 (50.8) | 1161 (49.8) |
| Age | 19-50 years | 1966 (65.7) | 2092 (54.6) | 951 (58.4) |
|  | 51-70 years | 877 (24.3) | 1151 (29.4) | 878 (31.5) |
|  | 71 + years | 445 (9.98) | 541 (15.9) | 292 (10.1) |
| Education | Low | 1003 (24.2) | 1076 (24.9) | 522 (19.5) |
|  | Medium | 1548 (46.8) | 1742 (49.1) | 972 (49.6) |
|  | High | 737 (29.0) | 966 (26.1) | 627 (30.9) |
| Area of residence | Major city | 2144 (72.9) | 2446 (72.2) | 1325 (75.1) |
|  | Regional remote | 618 (20.8) | 699 (19.4) | 465 (17.1) |
|  | Other | 526 (6.25) | 639 (8.44) | 331 (7.85) |

Values represent n and %. Data were adjusted for biomedical data survey weighting. The “Omitted sample based on only 1 x 24 hour recall” included individuals who were excluded based on only having completed one day of 24-hour dietary recall. The “Omitted sample based on other missing data” included eligible individuals with missing data for blood biomarkers, family history of diabetes, energy misreporting, physical activity, sedentary time and anthropometrics.
