# Supplementary material for: Association between diet quality, dietary patterns and cardiometabolic health in Australian adults: a cross-sectional study
Source: Nutr J. 2018 Feb 12;17:19. doi: 10.1186/s12937-018-0326-1 (PMC5809905; doi:10.1186/s12937-018-0326-1)
Supplement: Supplementary file 6 — Factor loadings for reduced rank regression dietary patterns. (DOCX 19 kb) [file 12937_2018_326_MOESM6_ESM.docx]

| Food groups | Factor loading | Variation explained |
| --- | --- | --- |
| DP-1 |  |  |
| Direct associations |  |  |
| Pome fruit (apples, pears, other pome fruit) | 0.23 | 9.42 |
| High fibre cereals and wholegrain pasta and brown rice | 0.22 | 9.17 |
| Dark, wholegrain, mixed grain breads, bread rolls | 0.22 | 8.56 |
| Nuts and seeds | 0.22 | 8.53 |
| Other vegetables | 0.21 | 8.08 |
| Carrot and root vegetables | 0.21 | 7.97 |
| Green and brassica vegetables | 0.20 | 7.23 |
| Other alternatives: soybeans or tofu, baked beans, other beans | 0.19 | 6.76 |
| Legumes | 0.18 | 5.68 |
| Peas and beans | 0.15 | 4.12 |
| Tropical and subtropical fruit | 0.14 | 3.46 |
| Citrus fruit | 0.14 | 3.73 |
| Tomato and tomato products | 0.13 | 3.22 |
| Stone fruit | 0.13 | 2.93 |
| Other fruit | 0.12 | 2.68 |
| Berry fruit | 0.10 | 1.66 |
| Processed cakes and pastries; Other puddings or desserts | 0.10 | 1.75 |
| Fish (raw, baked, roast, fried, grilled or BBQ’d) | 0.10 | 1.70 |
| Starchy vegetables | 0.09 | 1.39 |
| 2%, 1% or skim milk (including soy) and beverages (including flavoured milk) | 0.07 | 0.96 |
| Reduced, skim or no fat natural or flavoured yoghurt and reduced fat cheese | 0.07 | 0.96 |
| Non-wholegrain breakfast cereal and rice/pasta; quinoa; couscous; noodles | 0.05 | 0.53 |
| Wines | 0.05 | 0.40 |
| Soups: homemade, dry mix, canned | 0.02 | 0.08 |
| Chicken, turkey (trimmed, separable lean, lean, no skin) | 0.01 | 0.01 |
| Inverse associations |  |  |
| Fruit drinks, cordials and soft drinks | -0.24 | 10.69 |
| Regular fat milk (including soy) and beverages (including flavoured milk) | -0.24 | 10.54 |
| Cream or sour cream; Ice cream; custard; other dairy desserts | -0.22 | 9.17 |
| Chocolate | -0.21 | 8.41 |
| Non-wholegrain bread/bread rolls (including fried toast) | -0.20 | 7.72 |
| Processed/battered meat | -0.19 | 6.86 |
| Sugar products and dishes | -0.19 | 6.63 |
| Butter, animal-based solid fats and high fat dairy blends | -0.17 | 5.24 |
| Beers and ciders | -0.15 | 4.42 |
| Unsaturated margarine and oils | -0.11 | 0.89 |
| Snack foods: potato snacks, corn snacks | -0.09 | 1.52 |
| Regular fat natural or flavoured yoghurt (e.g. 2 or 3%) and reduced fat cheese | -0.09 | 0.94 |
| Other confectionary | -0.08 | 1.10 |
| Trimmed meat: Beef, veal, kangaroo, rabbit, venison, goat (<5% fat, 5-10% fat, trimmed, separable lean); Lamb (trimmed, separable lean); Pork (trimmed, separable lean) | -0.07 | 0.91 |
| Untrimmed meat: Beef, veal, kangaroo, rabbit, venison, goat, lamb, pork | -0.07 | 1.00 |
| Eggs | -0.05 | 0.46 |
| Pies, sausage roll or other savoury pastries; pizza | -0.04 | 4.28 |
| Fruit juice (including smoothie) | -0.03 | 0.18 |
| Sauces, dips and high-fat dressings | -0.03 | 0.13 |
| Dry or savoury biscuits, crisp bread, crackers | -0.03 | 0.05 |
| Fried vegetables (i.e. potato fries) | -0.02 | 0.05 |
| Tea, coffee, sports drinks, other beverages excluding fruit juices | -0.01 | 2.52 |
| DP-2 |  |  |
| Direct associations |  |  |
| Sugar products and dishes | 0.31 | 17.95 |
| Pome fruit (apples, pears, other pome fruit) | 0.28 | 18.68 |
| Tropical and subtropical fruit | 0.24 | 10.51 |
| Other fruit | 0.21 | 7.91 |
| Stone fruit | 0.20 | 7.44 |
| Fruit drinks, cordials and soft drinks | 0.18 | 14.44 |
| Cream or sour cream; Ice cream; custard; other dairy desserts | 0.16 | 12.12 |
| Citrus fruit | 0.16 | 6.67 |
| 2%, 1% or skim milk (including soy) and beverages (including flavoured milk) | 0.16 | 4.10 |
| Berry fruit | 0.14 | 3.87 |
| Fruit juice (including smoothie) | 0.13 | 2.20 |
| High fibre cereals and wholegrain pasta and brown rice | 0.12 | 10.81 |
| Carrot and root vegetables | 0.12 | 9.56 |
| Other confectionary | 0.12 | 2.77 |
| Peas and beans | 0.11 | 5.61 |
| Processed cakes and pastries; Other puddings or desserts | 0.10 | 2.96 |
| Reduced, skim or no fat natural or flavoured yoghurt and reduced fat cheese | 0.08 | 1.72 |
| Starchy vegetables | 0.04 | 1.61 |
| Regular fat natural or flavoured yoghurt (e.g. 2 or 3%) and reduced fat cheese | 0.04 | 1.58 |
| Other vegetables | 0.02 | 8.13 |
| Other alternatives: soybeans or tofu, baked beans, other beans | 0.02 | 6.80 |
| Green and brassica vegetables | 0.01 | 7.23 |
| Soups: homemade, dry mix, canned | 0.01 | 0.08 |
| Inverse associations |  |  |
| Beers and ciders | -0.30 | 15.20 |
| Wines | -0.30 | 11.21 |
| Regular fat milk (including soy) and beverages (including flavoured milk) | -0.24 | 13.49 |
| Chocolate | -0.21 | 12.71 |
| Fish (raw, baked, roast, fried, grilled or BBQ’d) | -0.18 | 5.36 |
| Non-wholegrain breakfast cereal and rice/pasta; quinoa; couscous; noodles | -0.19 | 4.57 |
| Non-wholegrain bread/bread rolls (including fried toast) | -0.15 | 10.21 |
| Trimmed meat: Beef, veal, kangaroo, rabbit, venison, goat (<5% fat, 5-10% fat, trimmed, separable lean); Lamb (trimmed, separable lean); Pork (trimmed, separable lean) | -0.15 | 3.44 |
| Eggs | -0.15 | 3.13 |
| Fried vegetables (i.e. potato fries) | -0.15 | 2.72 |
| Chicken, turkey (trimmed, separable lean, lean, no skin) | -0.14 | 2.23 |
| Unsaturated margarine and oils | -0.11 | 2.30 |
| Processed/battered meat | -0.10 | 7.99 |
| Snack foods: potato snacks, corn snacks | -0.08 | 2.21 |
| Untrimmed meat: Beef, veal, kangaroo, rabbit, venison, goat, lamb, pork | -0.08 | 1.76 |
| Sauces, dips and high-fat dressings | -0.08 | 0.89 |
| Nuts and seeds | -0.06 | 9.00 |
| Pies, sausage roll or other savoury pastries; pizza | -0.04 | 2.20 |
| Dark, wholegrain, mixed grain breads, bread rolls | -0.02 | 8.61 |
| Butter, animal-based solid fats and high fat dairy blends | -0.01 | 5.27 |
| Legumes | -0.01 | 5.68 |
| Tomato and tomato products | -0.01 | 3.22 |
| Tea, coffee, sports drinks, other beverages excluding fruit juices | -0.01 | 2.53 |
