# Supplementary material for: Association between diet quality, dietary patterns and cardiometabolic health in Australian adults: a cross-sectional study
Source: Nutr J. 2018 Feb 12;17:19. doi: 10.1186/s12937-018-0326-1 (PMC5809905; doi:10.1186/s12937-018-0326-1)
Supplement: Supplementary file 7 — Demographic and cardiometabolic characteristics of Australian adults across sex-specific tertiles (T) of each dietary pattern (n = 2121). (DOCX 21 kb) [file 12937_2018_326_MOESM7_ESM.docx]

**Table S6** Demographic and cardiometabolic characteristics of Australian adults across sex-specific tertiles (T) of each dietary pattern (n=2121)^1^

| Characteristic | Dietary pattern | | | *P*-trend^2^ |
| --- | --- | --- | --- | --- |
|  | T1 | T2 | T3 |  |
| DP-1 |  |  |  |  |
| Dietary pattern | -1.17 ± 0.05 | 0.06 ± 0.02 | 1.32 ± 0.05 | **<0.001** |
| Age, y | 43.9 ± 1.12 | 46.2 ± 1.14 | 49.0 ± 1.1 | **0.005** |
| Female, % | 50.0 | 49.4 | 50.2 | 0.98 |
| Highest level of education |  |  |  |  |
| Low | 22.0 | 17.4 | 19.1 | 0.42 |
| Medium | 49.3 | 52.6 | 46.8 |  |
| High | 28.7 | 30.0 | 34.1 |  |
| Smoking, % |  |  |  |  |
| Current smoker | 14.9 | 11.9 | 8.91 | 0.24 |
| Former smoker | 33.3 | 31.2 | 35.5 |  |
| Never smoked | 51.7 | 56.8 | 55.6 |  |
| Meet PA recommendations, % | 43.9 | 50.1 | 55.8 | **0.016** |
| Sedentary time, min/d | 359 ± 9.79 | 340 ± 10 | 331 ± 12 | 0.06 |
| HbA1c, mmol/mol | 35.2 ± 0.31 | 35.5 ± 0.33 | 36.3 ± 0.37 | **0.040** |
| Plasma glucose, mmol/L | 5.07 ± 0.03 | 5.04 ± 0.04 | 5.13 ± 0.04 | 0.35 |
| Total cholesterol, mmol/L | 5.04 ± 0.06 | 4.95 ± 0.05 | 5.00 ± 0.07 | 0.59 |
| HDL cholesterol, mmol/L | 1.34 ± 0.02 | 1.37 ± 0.02 | 1.33 ± 0.02 | 0.85 |
| LDL cholesterol, mmol/L | 3.12 ± 0.05 | 3.02 ± 0.05 | 3.09 ± 0.06 | 0.58 |
| Triglycerides, mmol/L | 1.24 ± 0.03 | 1.22 ± 0.03 | 1.24 ± 0.04 | 0.59 |
| Apolipoprotein B, g/L | 1.01 ± 0.02 | 0.98 ± 0.02 | 1.01 ± 0.02 | 0.93 |
| BMI, kg/m^2^ | 27.3 ± 0.34 | 26.8 ± 0.33 | 26.8 ± 0.30 | 0.43 |
| BMI category, % |  |  |  |  |
| Underweight/normal weight | 41.0 | 39.8 | 40.4 | 0.27 |
| Overweight | 30.5 | 37.4 | 37.3 |  |
| Obese | 28.6 | 22.8 | 22.3 |  |
| Waist circumference, cm | 91.7 ± 0.90 | 90.9 ± 0.88 | 91.2 ± 0.85 | 0.85 |
| Systolic blood pressure, mmHg | 119 ± 0.98 | 122 ± 1.08 | 122 ± 1.05 | **0.026** |
| Diastolic blood pressure, mmHg | 74.9 ± 0.72 | 76.5 ± 0.70 | 75.5 ± 0.63 | 0.53 |
| Overall risk score | -0.12 ± 0.04 | -0.12 ± 0.04 | -0.08 ± 0.04 | 0.56 |
| DP-2 |  |  |  |  |
| Dietary pattern | 0.08 ± 0.07 | -0.20 ± 0.05 | 0.15 ± 0.05 | 0.36 |
| Age, y | 44.8 ± 1.13 | 45.9 ± 1.27 | 48.4 ± 1.30 | 0.06 |
| Female, % | 49.8 | 49.7 | 50.0 | 1.00 |
| Highest level of education |  |  |  |  |
| Low | 18.7 | 19.0 | 20.8 | 0.89 |
| Medium | 49.9 | 48.6 | 50.3 |  |
| High | 31.4 | 32.4 | 28.9 |  |
| Smoking, % |  |  |  |  |
| Current smoker | 19.0 | 7.67 | 9.16 | **<0.001** |
| Former smoker | 30.4 | 34.1 | 35.7 |  |
| Never smoked | 50.7 | 58.3 | 55.2 |  |
| Meet PA recommendations, % | 52.4 | 49.3 | 48.8 | 0.68 |
| Sedentary time, min/d | 353 ± 10.1 | 333 ± 11.2 | 343 ± 10.6 | 0.45 |
| HbA1c, mmol/mol | 35.5 ± 0.30 | 35.3 ± 0.34 | 36.1 ± 0.32 | 0.12 |
| Plasma glucose, mmol/L | 5.09 ± 0.35 | 5.06 ± 0.04 | 5.10 ± 0.04 | 0.81 |
| Total cholesterol, mmol/L | 5.08 ± 0.07 | 4.89 ± 0.05 | 5.03 ± 0.06 | 0.76 |
| HDL cholesterol, mmol/L | 1.39 ± 0.02 | 1.34 ± 0.02 | 1.31 ± 0.02 | **0.026** |
| LDL cholesterol, mmol/L | 3.10 ± 0.06 | 3.00 ± 0.04 | 3.14 ± 0.06 | 0.58 |
| Triglycerides, mmol/L | 1.26 ± 0.04 | 1.18 ± 0.04 | 1.26 ± 0.04 | 0.98 |
| Apolipoprotein B, g/L | 1.00 ± 0.02 | 0.97 ± 0.02 | 1.02 ± 0.02 | 0.52 |
| BMI, kg/m^2^ | 26.4 ± 0.31 | 27.2 ± 0.41 | 27.3 ± 0.34 | 0.10 |
| BMI category, % |  |  |  |  |
| Underweight/normal weight | 44.7 | 40.2 | 36.3 | 0.11 |
| Overweight | 34.6 | 31.5 | 39.1 |  |
| Obese | 20.7 | 28.3 | 24.6 |  |
| Waist circumference, cm | 90.3 ± 0.89 | 91.6 ± 1.06 | 92.1 ± 0.82 | 0.14 |
| Systolic blood pressure, mmHg | 119 ± 1.33 | 122.3 ± 1.03 | 121 ± 1.04 | 0.37 |
| Diastolic blood pressure, mmHg | 75.8 ± 0.83 | 76.5 ± 0.55 | 74.6 ± 0.70 | 0.33 |
| Overall risk score | -0.15 ± 0.04 | -0.11 ± 0.05 | -0.07 ± 0.04 | 0.22 |

1, BMI, body mass index. DP, dietary pattern; Education: low (completed some high-school or less), medium (completed high-school or completed some high-school and/or certificate/diploma) and high (having a tertiary qualification). BMI category: underweight/ normal weight (BMI<25 kg/m^2^), overweight (25≤BMI<30 kg/m^2^), obese (BMI≥30 kg/m^2^); Overall cardiometabolic risk score was based on WC, TAG, HDL-cholesterol, blood pressure (average blood pressure was used as an index for systolic and diastolic blood pressure), and fasting plasma glucose based on an established methodology [[25](file:///L:\Endeavour%20papers\Study%201b%20and%202b%20datalabs%202\Manuscript\Nutrition%20Journal\Livingstone_Manuscript.docx#_ENREF_25)]. Values represent mean ± SE after adjustment for survey weighting. Where transformed for regression analyses, values represent exponentiated geometric mean ± SE;

2, Linear regression analyses (continuous variables) and χ^2^ (categorical variables) were used to test for trends across tertiles.
